# Utilization and outcomes of cervical cancer screening services in Harare City, 2012–2016: a secondary data analysis

**DOI:** 10.1186/s12913-019-4314-8

**Published:** 2019-07-05

**Authors:** Christine Gabaza, Prosper Chonzi, Addmore Chadambuka, Gerald Shambira, Tsitsi Patience Juru, Notion Tafara Gombe, Peter Nsubuga, Mufuta Tshimanga

**Affiliations:** 1 Department of Community Medicine, University of Zimbabwe, Harare, Zimbabwe; 2 Department of Health, Harare city Council, Harare, Zimbabwe; 3 African Field Epidemiolog, Network ( AFENET), Kampala, Uganda

**Keywords:** Cervical cancer, VIAC, Cancer screening, Zimbabwe

## Abstract

**Background:**

Visual inspection with acetic acid and cervicography (VIAC) is a method used to screen for cervical cancer. VIAC can be used as part of a “see and treat” strategy. Nine Harare city council health facilities offer VIAC free of charge with the aim of reducing morbidity and mortality from cervical cancer. Between 2014 and 2016, the number of women utilising VIAC dropped by 35%. We analysed records of clients who utilise VIAC at Harare city health facilities to characterise women accessing VIAC and their outcomes to make recommendations for improving the services.

**Methods:**

We conducted a descriptive cross-sectional study using data collected for the Harare city VIAC program. We analysed all records of clients who utilised VIAC services at nine Harare city health facilities from 1 May 2012 to 31 December 2016.

**Results:**

We analysed 46,217 records, the median age of the clients was 34 years [Q_1_ = 27: Q_3_ = 42]. Of the 46,217 clients screened, 3001 (6.5%) were VIAC positive, and 512 (1.1%) had suspicious of cancer lesions. The prevalence of VIAC positive ranged from 58 to 74 per 1000-screened clients over the 5 years. The prevalence of suspected cancer ranged from 9 to 14 per 1000-screened clients, and there was a general decrease in the prevalence between 2012 and 2016. Of the 3513 clients with VIAC positive or had suspicious of cancer lesions, 2090 (74.1%) did not receive treatment at the site where the screening took place.

**Conclusion:**

The majority of women who are accessing VIAC services in Harare are middle-aged, multiparous and married women. There is a treatment gap at most of the VIAC centres such that clients are referred to other centres for management. The objective of “see and treat” is not being realised.

## Background

Worldwide, cervical cancer is the fourth most common malignancy in women after breast, lung and colorectal cancer [1]. About 528,000 cases of cervical cancer occur annually, and over 250,000 women die from the malignancy. The majority (87%) of the deaths occur in developing countries [1]. In sub-Saharan Africa, cervical cancer ranks second among the common malignancies in women, with an estimated 94,000 cases occurring every year [1]. In Zimbabwe, cervical cancer is the most common and the leading cause of death from malignancies in women. In 2016, it accounted for 33% of the cancer burden in women and 12% of deaths from malignancies in women [2]. Without urgent attention, deaths due to cervical cancer are projected to continue to increase [3].

Cervical cancer is one of the few malignancies that is preventable. A 10 to 20 year lag between the pre-cancer and the invasive stages offers an opportunity to screen, detect and treat the disease before its progression to cancer [4]. Methods of screening include Papanicolaou (Pap) smear, Visual Inspection with Acetic Acid and Cervicography (VIAC), and human papillomavirus deoxyribonucleic (HPV DNA) testing [4–6]. In Zimbabwe, VIAC is the method used for screening mostly in the public sector while both VIAC and PAP smears are offered in the private sector [7]. Zimbabwe has also recently started to vaccinate girls between 10 and 14 years against HPV.

VIAC entails taking pictures of the cervix after applying 3–5% acetic acid to the cervix [5]. A VIAC result is negative if the cervical tissue remains normal while the appearance of white areas indicates a positive result. A suspicious of cancer result is when the lesions appear very thick, and this suggests invasive cancer [3]. VIAC is a cost-effective method that can be performed by a wide range of trained health workers such as doctors, midwives and nurses [8].

Zimbabwe adopted VIAC in 2012; it is offered at public health facilities free of charge [8]. At these facilities, the “see and treat” approach is implemented. “See and treat” means that treatment is offered immediately to clients who screen VIAC positive. Existing treatment methods for the “see and treat” strategy include cryotherapy, loop electrosurgical excision procedure (LEEP) and cauterisation [9]. For those with suspicious of cancer lesions, clients are referred to the next level of care for biopsies.

Harare, the capital city of Zimbabwe, had an estimated 2018 population of 1.5 million (projected from the 2012 census) of which almost 950,000 (64%) are women aged 15–64 years [10]. The Harare city health department started offering VIAC in 2012 and has a target of screening 10,000 women annually. Between 2014 and 2016, the number of women utilising VIAC dropped by 35% (i.e., from 11,101 in 2014 to 7,209 in 2016). The health department made efforts to increase VIAC uptake through educating women as they visit health facilities for other services (e.g., postnatal care, outpatient visits, and HIV clinics) and conducting community campaigns. We analysed records of clients who utilise VIAC at Harare city health facilities to characterise women accessing VIAC and their outcomes to make recommendations for improving the services.

## Methods

### Study type

We conducted a descriptive cross-sectional study using data collected for the VIAC program. Our outcomes of interest were VIAC results, which were positive, negative or suspicious of cancer. Data collection for this study included records from 1 May 2012 to 31 December 2016.

### Study setting

We conducted this study in Harare city council health facilities that offer VIAC services. Harare is the capital of Zimbabwe. The city council provide health care services, complemented by private healthcare providers. Harare city has nine districts, and each district has one council centre offering VIAC services. These include the following clinics; Edith Opperman, Mabvuku, Highfields, Glen Norah, Budiriro, Warren Park, Marlborough, Hatcliffe and Wilkins hospital. These facilities except Wilkins hospital, offer primary health services to Harare city residents. Wilkins hospital is a referral centre for infectious diseases, and it serves clients that are not limited to residents of Harare.

### Data sources and types

We used data collected from clients who received VIAC services at Harare city health facilities from 1 May 2012 to 31 December 2016. One record represents one client who received VIAC services. Clinicians collect the data when clients visit the VIAC clinics. The primary purpose for collecting the data is for monitoring and evaluating the VIAC program. Variables captured in this dataset include age, marital status, parity, HIV status, contraceptive use, the age of sexual debut, number of sexual partners, history of sexually transmitted infections, presenting complaints, VIAC results in treatment given, and type of visit (i.e., first visit or repeat visit). Clinicians capture these variables in hard copy registers then transfer the information into electronic records in Epi Info 3 or 7™ (United States Centres for Disease Control and Prevention).

### Study unit

An individual client record was the study unit.

### Data management

We analysed all 46,217 electronic records of clients that received VIAC services from 1 May 2012 to 31 December 2016. The primary data sources that we used were electronic datasets in Epi Info from the nine VIAC centres in Harare city. Six centres use Epi Info version 3; three use Epi Info version 7. We collected the electronic datasets from the nine health facilities and aggregated them into Epi Info 7™. To have uniform variables, we re-coded variables of interest from the nine datasets to 12 uniform variables. The variables we defined were; age, marital status, type of visit, the presence of gynaecological symptoms, age at sexual debut, parity, the total number of lifetime sexual partners, condom use, lifetime history of sexually transmitted infections, VIAC results and treatment that was given. We checked the data for missing information and inconsistencies. We used hard copy VIAC registers from the clinics to update missing information and to correct data inconsistencies.

### Data analysis

We used Epi Info 7™ to generate frequencies, means and proportions. We used Microsoft Excel to generate graphs and to perform simple linear regression for trends. The results were considered statistically significant if the *p*-value for the model was ≤0.04. We analysed all available records. If a record had missing information on a specific variable, we excluded that record in the analysis of the missing variable.

### Permission and ethical considerations

We sought permission to carry out this study from the Harare City ethical review board, and Health Studies Office (HSO) in the Ministry of Health and Child Care. We maintained confidentiality by not including client names or any personal identifiers in capturing data, analysis and reporting.

## Results

### Socio-demographic and sexual behaviour characteristics of clients

From 1 May 2012 to 31 December 2016, the recorded women screened for cervical cancer were 46,217. All the records had a completion rate of over 80% on the variables of interest, and we analysed all the records.

All 46,217 clients had ages recorded, 30,542 (66.0%) were in the 21–40 year age group. The median age of the clients was 34 years [Q_1_ = 27: Q_3_ = 42]. Most of the clients were married 37,608 (81.4%). Of the 46,206 clients with a recorded number of lifetime sexual partners, 17,352 (37.6%) had ≥2. Ten per cent of 45,575 clients reported always using condoms. Among 46,180 clients with recorded HIV status, 9,651 (20.9%) were positive and 1,336 (2.6%) had unknown HIV status (Table 1).

**Table 1:** Socio-demographic and sexual behaviour characteristics of clients presenting for viac services, Harare city, 2012–2016

Variable	Category	Frequency	
		Number	Percent
Age group(*n* = 46,217)	15–20	2252	4.9
	21–30	15,505	33.5
	31–40	15,036	32.5
	41–50	7797	16.9
	50+	5627	12.2
Median age = 34(Q_1_ = 27; Q_3_ = 42)			
Marital status (n = 46,217)	Single	2093	4.5
	Married	37,608	81.4
	Divorced/Separated	2107	4.6
	Widowed	4409	9.4
Parity (n = 46,124)	0	3344	7.3
	1–4	37,549	81.4
	≥5	5231	11.3
	missing^a^	93	
Median number of children = 2(Q_1_ = 2; Q_3_ = 3)			
Age at sexual debut (n = 46,217)	< 16	3276	7.1
	16–17	7511	16.3
	≥18	35,430	76.6
Median age at sexual debut = 19 (Q1 = 18; Q_3_ = 21)			
Number of lifetime sexual partners (n = 46,206)	1	28,854	62.4
	≥2	17,352	37.6
	missing^a^	11	
Median number of lifetime sexual partners = 1(Q_1_ = 1;Q_2_ = 2)			
Condom use (*n* = 45,575)	Always	4644	10.1
	Sometimes	16,084	35.2
	Never	25029	54.7
	missing^a^	642	
History of a STI (*n* = 42,217)	Yes	9562	22.6
HIV status (n = 46,180)	Unknown	1336	2.9
	Negative	35193	76.2
	Positive	9651	20.9
	missing^a^	37	

^a^= missing observations were not included in calculating proportions

### VIAC outcomes by demographic characteristics, sexual behaviour and HIV status

Of the 46,217 clients screened, 3001 (6.5%) were VIAC positive, and 512 (1.1%) had suspicious of cancer lesions. VIAC positivity did not differ significantly across the nine clinics (i.e., 6.2–6.8%). Comparing VIAC outcomes by age groups, the 31–40 year age group had the highest proportion with VIAC positive (8.1%), followed by the 21–30 year age group (6.7%). Among 9,651 HIV positive clients, 1,311 (13.6%) were VIAC positive, and 206 (2.1%) had suspicious of cancer lesions. Lesions that were assessed as suspicious of cancer were highest among those > 50 years (i.e., 173/5627 (3.1%)).

Comparing VIAC outcomes by marital status, the group with the highest VIAC positives was single women [i.e. 214/2093 (10.2%)] followed by the separated or divorced (i.e. 204/2107 (9.7%). The widowed group had the highest proportion of suspicious of cancer lesions (3.1%). Of the 4,644 clients who always used condoms, 519 (11.1%) were VIAC positive while 2430 (5.6%) of 41,113 clients who did not always used condoms were VIAC positive (Table 2).

**Table 2:** VIAC Outcomes by demographic characteristics, sexual behaviour, and hiv status, Harare City 2012–2016

Variable	Total screened	VIAC positive		Suspicious of cancer	
		Number	Percent	Number	Percent
Age group	n = 46,217				
15–20	2252	118	5.2	5	0.2
21–30	15,505	1036	6.7	59	0.4
31–40	15,036	1216	8.1	145	1.0
41–50	7797	501	6.4	130	1.7
50+	5627	130	2.3	173	3.1
Marital status	n = 46,217				
Single	2093	214	10.2	26	1.2
Married	37,608	2271	6.0	307	0.8
Widowed	4409	312	7.1	137	3.1
Separated/divorced	2107	204	9.7	42	2.0
Age at sexual debut	n = 46,167				
< 16	3226	250	7.6	74	2.3
16–17	7511	545	7.3	103	1.4
≥ 18	35,430	2206	6.2	335	0.9
Number of lifetime sexual partners n = 46,184					
1	28,836	1490	5.2	278	1.0
> 1	17,348	1511	8.7	234	1.3
Always use Condom	n = 45,757				
Yes	4644	519	11.2	50	1.1
No	41113	2430	5.6	459	1.1
History of a STI	n = 46,195				
Yes	9556	886	9.3	143	1.5
No	36,639	2115	5.8	369	1.0
HIV status	n = 46,180				
Positive	9651	1311	13.6	206	2.1
Unknown	1336	54	4.0	25	1.9
Negative	35,193	1627	4.6	218	0.8

### Trends of VIAC positive and suspected cancer among screened clients 2012–2016

**Fig. 1:**
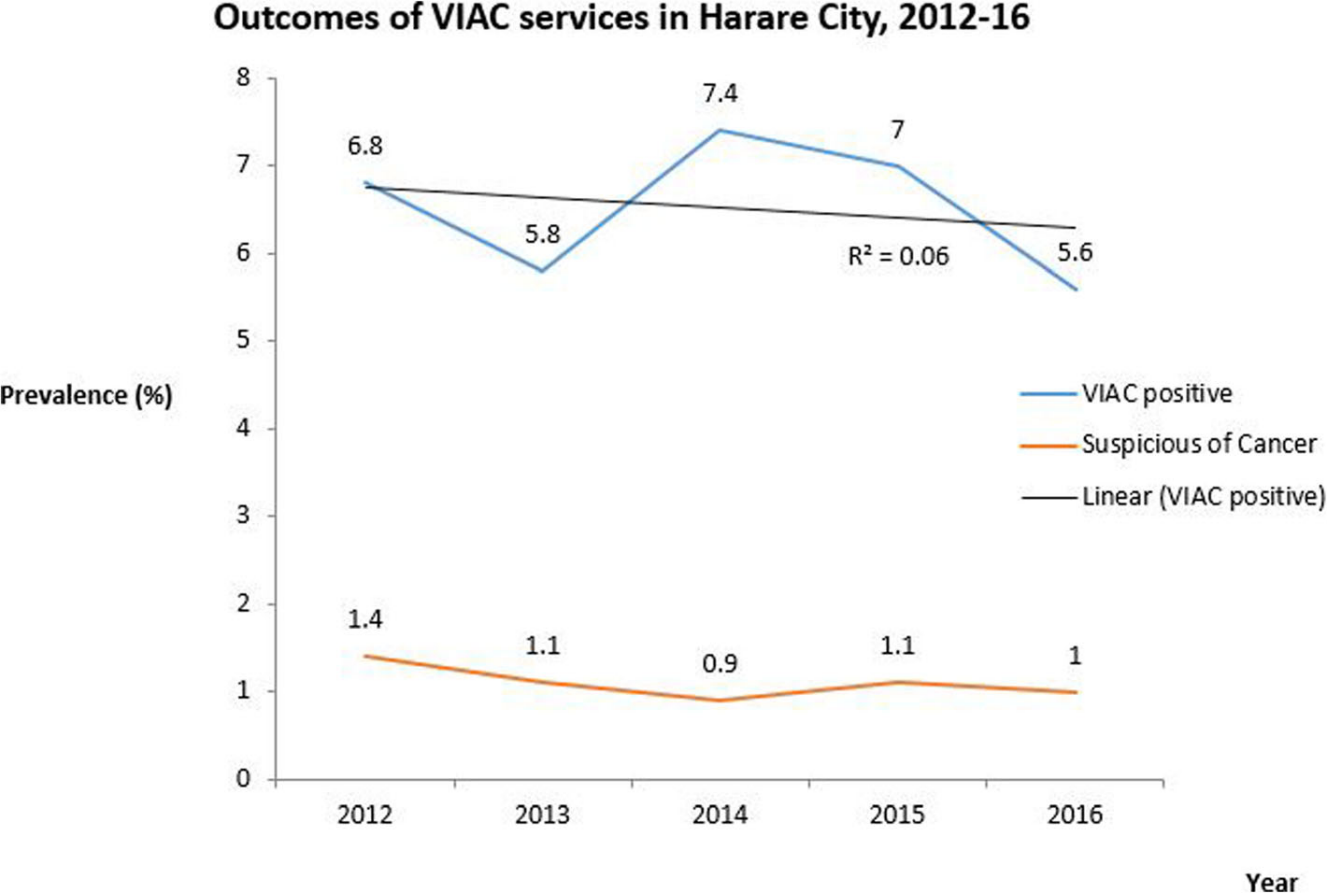
Prevalence of precancerous cervical lesions by year among VIAC clients, Harare city, 2012–2016

The prevalence of VIAC positive ranged from 5.8 to 7.4% over the 5 years. There was a decrease in the prevalence from 6.8 to 5.8%between 2012 and 2013 then an increase to a peak of 7.4% in 2014. The prevalence then decreased to 5.6% in 2016. We did not find any significant linear trends of VIAC prevalence over the 5 years (R^2^ = 0.06, *p* = 0.69). The prevalence of suspected cancer ranged from 0.9 to 1.4% and showed a general decline from 2012 to 2016 (Fig. 1).

### Management that was given to clients with VIAC positive or those with lesions that were assesed as suspicious of cancer

**Fig. 2:**
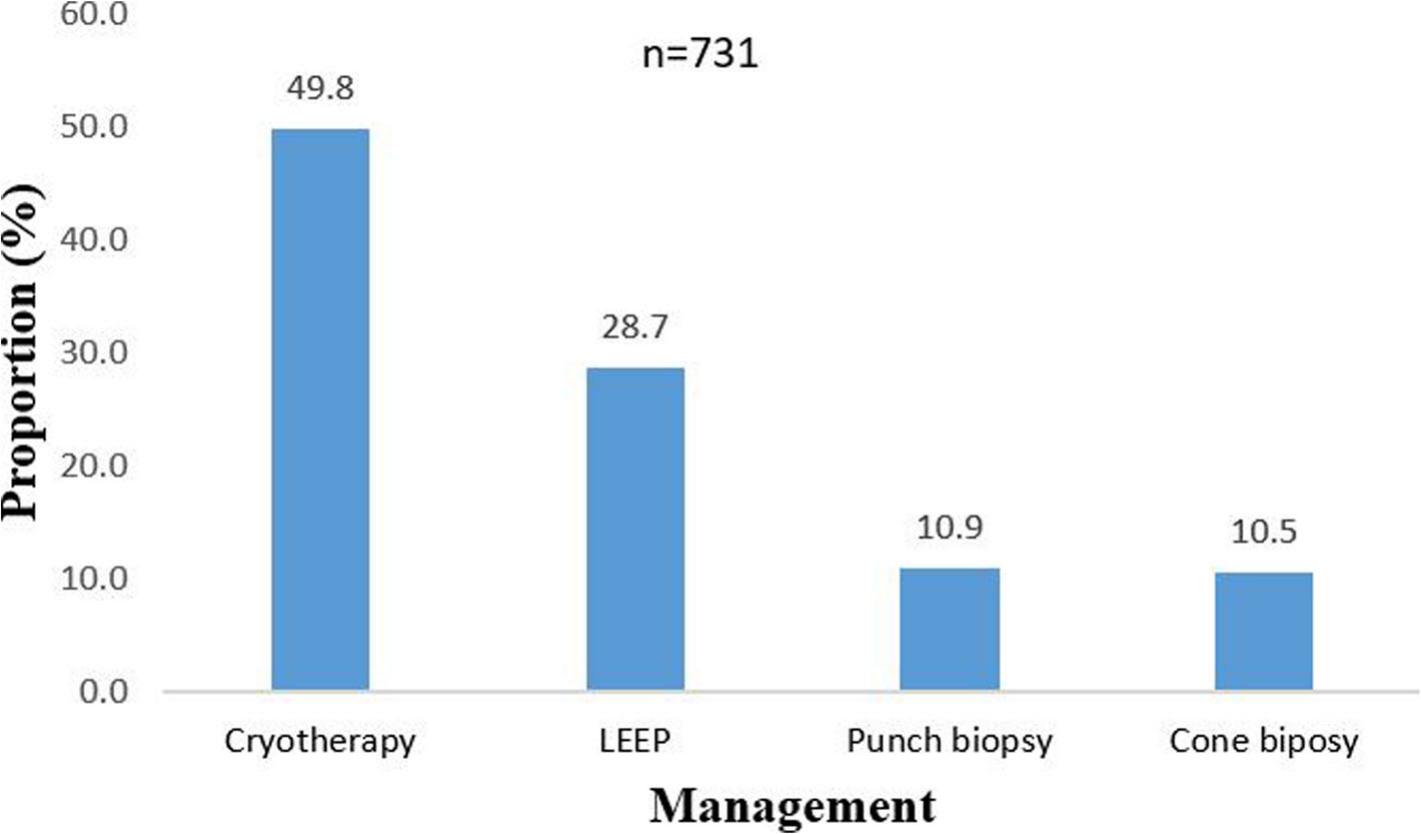
Proportions of clients presenting for VIAC with gynaecological symptoms Harare City, 2012–2016

Four out of the nine VIAC centres offer VIAC treatment at the same site. One clinician does LEEP at one of the sites. Of the 3513 clients who were VIAC positive or had suspicious of cancer lesions, 2,821 (80.3%) had treatment offered recorded. Of these, 2,090 (74.1%) did not receive treatment at the same site where the screening took place. These clients were referred to other facilities for treatment. Of the 867 clients treated at the same site where screening was done, 364 (49.8%) had cryotherapy, 210 (28.7%) had LEEP, 80 (10.9%) had punch biopsies, and 77 (10.5%) had cone biopsies (Fig. 2). All 867 clients who were treated at the same site where screening was done were treated on the same day.

### Trends of visits for VIAC 2012–2016

**Fig. 3:**
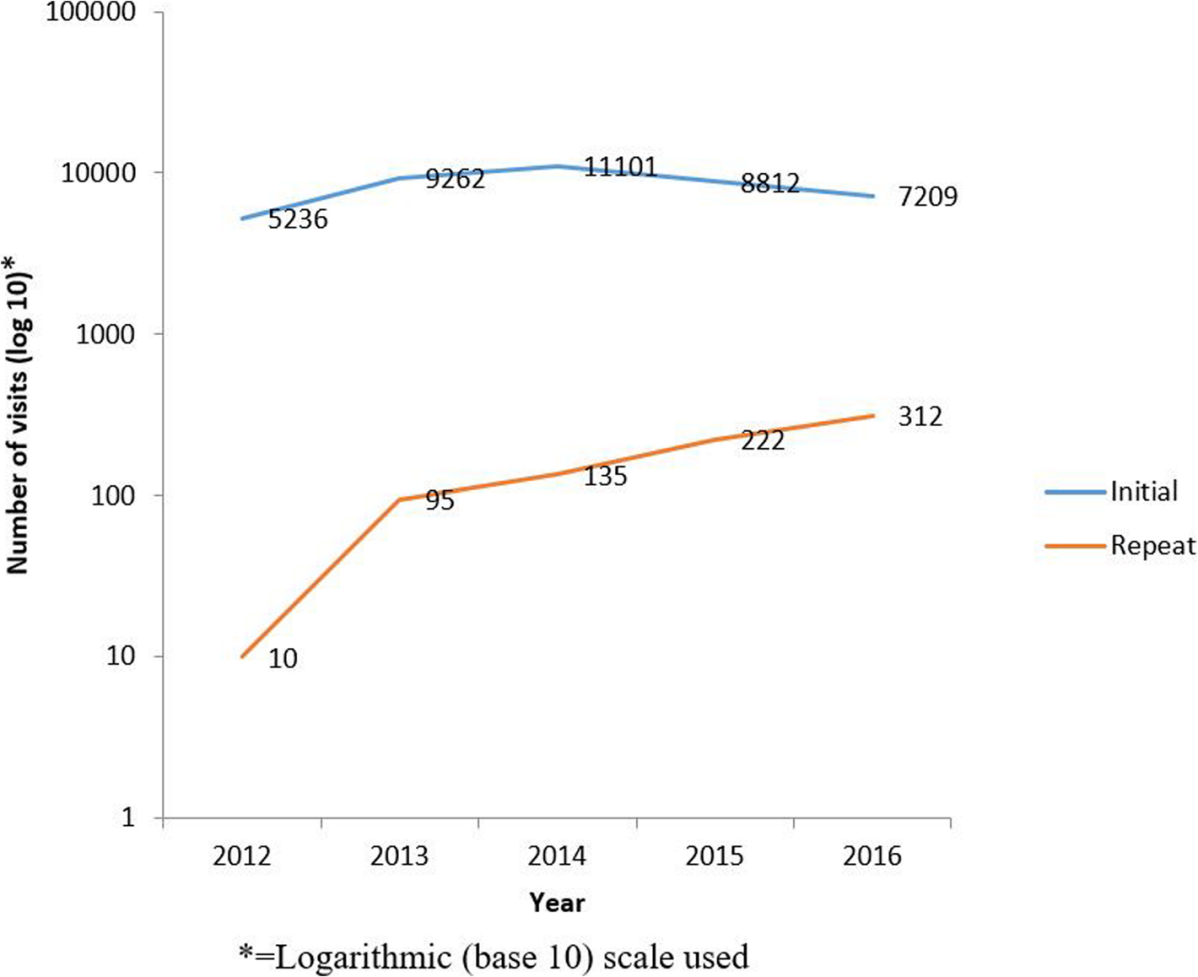
Management given to clients with VIAC positive or suspicious of cancer results, Harare City 2012–2016

The number of initial visits for VIAC services increased from 5,236 in 2012 and peaked at 11,101 in 2014. From 2014, the number of initial visits for VIAC services decreased to 7,209 in 2016. Repeat visits for VIAC showed a general increase from 10 in 2012 to 312 in 2016 (Fig. 3).

## Discussion

We found that among screened women the prevalence of VIAC positive was 6.5%. Studies conducted in South Africa, Angola and Mozambique, reported almost similar VIAC positive prevalence (6.6–8%) to what we found in our study [11–13]. In these countries, the burden of cervical cancer is comparable to Zimbabwe; hence, the burden of pre-cancerous lesions may also be similar. Another Zimbabwean study Fallala and Mash in 2015 reported VIAC positive prevalence of 10.8% [14]. Paharm et al. in their study in Zambia reported a much higher prevalence than what we found (i.e., 20%) [15]. The Zimbabwean and Zambian studies had a higher proportion of HIV positive women compared to ours (i.e., 52.5 and 28% respectively) and this may explain the high VIAC prevalence.

HIV prevalence of 20.9% among the VIAC screened women is high compared to the national average of 16.7% that was reported in the Zimbabwe Population-Based Impact Study (ZIMPHIA) [16]. This finding may be because HIV positive women have greater interaction with healthcare providers hence more opportunities to gain awareness and access to VIAC services. This finding may also indicate that the VIAC services in Harare City are also reaching more of the women that are at risk of developing cervical cancer. We also found that some women who were screened had unknown HIV statuses. These women were referred for HIV testing, but no verification was made to ensure that the clients accessed HIV testing services. Services like VIAC can be an opportunity to identify women who do not know their HIV statuses in order to complement efforts towards achieving the first 90 target (i.e., 90% of people living with HIV should know their status by 2010) of the United Nations Programme on HIV/AIDS (UNAIDS) [17].

The majority of women who utilised VIAC services in Harare city were married. Kahesa et al. and Ncube et al. in their studies found out that married women were more likely to use cervical cancer screening services [18, 19]. Social support from one’s spouse may be the reason for this high uptake. Conversely, Mupepi et al. reported that being married was associated with low uptake of cervical cancer screening [20]. This finding indicates the need to engage men in promoting uptake of cervical cancer screening services.

The finding that VIAC positive prevalence was higher in those who always used condoms compared to those who did not is contrary to other results. Fukuchi et al., in a Zimbabwean study, found out that infrequent condom use was associated with precancerous cervical lesions [21]. Another study by Lam et al. reported similar results [22]. The reason for this contrary finding may be that in our study, 65% of the clients who always-used condoms were HIV positive. HIV infection is a known risk factor for developing cervical precancerous lesions [23, 24]. Hence the finding in our study was somewhat confounded by HIV status.

The majority of the clients who were VIAC positive or had suspected cancer were referred to another centre for treatment. Out of the nine centres that are offering VIAC services, only four offer treatment for VIAC positive clients and there is one clinician offering LEEP. Reffering clients to other sites for treatment poses a challenge, as some of the clients may not access the referral centres resulting in missed opportunities for treating these women. These five centres did not have the equipment for managing VIAC positive clients and had shortages of consumables (e.g., nitrous oxide) required for treating the clients run out of stock. As a result they were not able to offer treatment as needed according to the “see and treat” approach. We hypothesize that such stock out of VIAC consumables and lack of equipment to manage VIAC positive clients may be the reason why clients are no longer coming for VIAC services.

Our study had a few limitations to the generalisation of our findings beyond our specific context. The women who use VIAC services at Harare city centres are not limited to Harare residents. Hence, coverage for the VIAC service in Harare cannot be determined from this study. The second limitation is that we cannot completely rule out that some clients who utilised VIAC services were not recorded therefore the dataset may not have been complete. However, we feel that our study was able to characterise women who are accessing VIAC services in Harare city.

## Conclusion

In conclusion, the majority of women who are accessing VIAC services in Harare are middle-aged, multiparous and married women. There is a treatment gap at most of the VIAC centres such that clients are referred to other centres for management. Therefore, the “see and treat” approach is not being operationalised at all the VIAC centres.

We recommend capacitation of the remaining five centres so that screening and treatment are offered at the same site. We also recommended follow up means be created for those who are identified as suspicious of cancer to ensure that they access services at the next referral centre. Following discussion of our finding with health workers at the VIAC centres, mechanisms to link clients with unknown HIV status were created at all sites. If a client with unknown HIV status is identified, instead of being referred for HIV testing, the service is now offered at the VIAC clinic.

## Data Availability

The data that support the findings of this study are available from the Ministry of Health & Child Care Zimbabwe, but restrictions applies to the availability of these data. Data are however available from the authors upon reasonable request and with permission from Ministry of Health Child Care Zimbabwe.
